# Questioning evidence-based practice in physiotherapy: a philosophical reflection. *The example of physiotherapy with musicians*

**DOI:** 10.3389/fpsyg.2024.1427648

**Published:** 2024-08-20

**Authors:** Céleste Rousseau, Barbara Stiegler

**Affiliations:** ^1^Centre Européen d’Enseignement en Rééducation et Réhabilitation Fonctionnelle, Saint-Denis, France; ^2^UMR(U) 4574 Sciences, Philosophies, Humanités, Université Bordeaux Montaigne, Pessac, France

**Keywords:** evidence based practice (EBP), musicians’ health, physiotherapy, ethics, performance

## Abstract

**Introduction:**

In the late 1990s, evidence-based medicine (EBM) emerged, emphasizing the conscientious use of current best evidence in medicine and ensuring the best care for each individual. Thus, applying evidence-based practice (EBP) in physiotherapy is complex due to sparse research, distinct challenges, and unsuitable tools. This is particularly relevant considering musicians’ health since the literature is very limited.

**Part I:**

The first part advocates for EBP and explain how it ensures both the best care and adherence to ethical principles (beneficence, non-maleficence, respect for autonomy, and justice), for both caregivers and patients.

**Part II:**

The second part discusses the common pitfalls often encountered by healthcare professionals, and especially as physiotherapists for musicians, when applying evidence at the patient’s bedside, again from both perspectives, healthcare professionals and patients.

**Part III:**

Finally, this third part aims to open the discussion by considering various perspectives, such as values-based practice or the importance of qualitative research, to reshape EBP in physiotherapy.

**Conclusion:**

This work highlights the prevalent existence of *grey zones* encountered by healthcare practitioners with musicians. While conducting more research to help understand them, physiotherapists must learn to navigate these waters.

## Introduction: evidence-based practice with musicians, a philosophical issue

Evidence-based medicine was defined in the late 1990s as the “conscientious, explicit, and judicious use of current best evidence in making decisions about the care of individual patients” (Sackett et al., 1996). Paramedical professions adopted this approach, and to avoid excluding these professions, we prefer the use of “evidence-based practice” instead of medicine, which now seems inappropriate and limited.

In his *princeps* paper (Sackett et al., 1996), David Sackett states that the philosophical foundations of the evidence-based approach date back to at least mid-19th century Paris, likely referring to French positivism and experimental medicine. The Evidence-Based Medicine Working Group (1992) has described EBM as being a “paradigm shift,” clearly referring to Kuhn (Kuhn, 1983) and his conception of changes in scientific paradigm, discussing the importance of the “development of clinical instincts” (regarding what cannot be tested) and the understanding of “certain rules of evidence,” important for the well-read and implementation of the proof with our patients. Evidence-based practice (EBP) was therefore defined under the famous shape of the following tryptic: the combination of research evidence, clinical expertise and patient preferences (Haynes et al., 2002). Since then, this tryptical definition has been reshuffled due to much criticism, mainly the lack of a clear relationship between the three circles which are theoretically of equal value (Bizouarn, 2019).

Moreover, five principles have been described, highlighting the relationship between EBM and our daily clinical practice (i.e., second principle, “secondly, the clinical problem – rather than habits or protocols – should determine the type of evidence to be sought”) (Davidoff et al., 1995). The connection between EBP and clinical practice requires deeper description. In *The Birth of Clinic* (Naissance de la Clinique), Michel Foucault (French philosopher, 1926–1984) describes the different paradigms that followed one another regarding medicine and its main object: illness. Foucault described how the medical sight on the individual has permitted “a scientifically structured discourse” by removing “the old Aristotelian forbidding,” and thought the “singular colloque” of the care relationship to be one with an asymmetrical power balance, using sarcasm and phrases like the “the doctor-patient couple” (Foucault, 2003).

If EBP promoters consider it a foundational necessity in clinical practice, this contrasts with Maël Lemoine’s (contemporary French philosopher) view. He argues that the clinic should be considered as a very specific procedure to medicine, binding observations (joining Foucault here) and explanations. In that respect, the clinic cannot be merely equated to proof integration. In his work “The disunity of medicine,” Lemoine explains how medicine consists of different fields not specific to medicine (i.e., physics, chemistry, biology), and therefore, different explanations coexist (Lemoine, 2011). For Lemoine, the only specific approach in medicine is the clinic, which cannot be confused with the traditional evidence-based approach since it is only a statistical explanation of medicine, or with EBP since it is only an “evaluation approach of one aspect of clinical practice (therapeutic decision), based on statistical methods which in medicine belong rather to the epidemiological subfield” (Lemoine, 2011).

The use of scientific evidence in medicine is not new. In the 19th century, after the decline of anatamo-pathological medicine, Claude Bernard’s approach of experimental medicine experienced an impressive rise. And in the mid-20^th^ century, Hill theorized the randomized controlled trial (RCT) (Hill, 2011), which is now a pillar of the evidence hierarchy. EBP classifies and ranks evidence based on quality and inherent risk of bias: at the top of the pyramid, we find RCTs, systematic reviews, meta-analyses and overviews (Howick et al., 2011). However, while RCT minimizes bias through randomization and blinding, it does not allow researchers to open the “black box” of underlying physio-pathological mechanisms (Rogers and Hutchison, 2017). This may explain why the external validity of RCTs, and their applicability to clinical practice in populations or conditions outside of those tested, is disputable. This element of external validity and applicability is what makes the treatment of musicians using EBP difficult. Can we really apply exercises described for swimmers to violinists lacking scapular stabilization (Tawde et al., 2016) or understand low back pain in pianists by comparing them to office workers?

Moreover, this primarily concerns the clinical questions which have been investigated in research. How is it possible to apply evidence with double bassists while specific research on this instrument is scarce, if not non-existent (Levenderis and Rennie-Salonen, 2022)? One might also wonder about the relevance of the tools used in many studies: is the Rated Perceived Exertion Scale the most appropriate to evaluate musical performance (McCrary et al., 2016), or Rapid Entire Body Assessment for investigating playing postures? (Valenzuela-Gómez et al., 2020). To this day, to the best of our current knowledge, no one has undertaken to reflect on evidence-based practice at the musicians’ bedside, by mobilizing both scientific references and philosophical insights to better understand what is at stake.

Thus, here is the question we are asking: in the specific context of musician’s health, does EBP provide the best guarantee of care, or is it a chimera (an unattainable ideal) or a straightjacket?

### Methodological considerations

This philosophical research work is conceived in three parts. Two contradictory theses about the implementation of evidence-based practice and its pragmatic approach in the context of musicians’ physiotherapy are presented in part one and two, considering treatment and prevention. The third part consists of a synthesis of these two opposing arguments and provides perspectives for both clinicians and researchers, who may recognize themselves in the question raised throughout this reflective paper. This cannot be considered as a standardized literature review but rather as a cross-reading of a selected *corpus* consisting of scientific research and philosophical works.

## Part I: evidence-based practice, a guarantee for best healthcare

### For professionals, providing the best healthcare

EBP evaluates the best available evidence on treatments or diagnostic tools and, depending on the pathology and individual case, seeks to provide the best solution for healthcare practitioners. This is a reassuring approach for caregivers, particularly for younger caregivers who cannot rely on their own clinical expertise. In that respect, the difference between knowledge and practice must be emphasized – knowing the evidence is not enough to know how to apply them. This is where the clinical experience enters the scene and how the two circles of the triptych overlap. This is apparent when examining trained professionals in the McKenzie method, who have more reliable abilities to classify low back pain patients than those who are not trained (Garcia et al., 2018).

In addition, being familiar with best and latest scientific evidence also means being able to communicate them to our patients and argue with our peers. This competence allows to effectively explain to other healthcare practitioners who may advise injured musicians to abruptly interrupt their instrument practice, that this sudden stop may not be beneficial in the long term (Stanhope and Weinstein, 2021).

Finally, basing clinical practice on evidence requires an acknowledgement of, and adherence to, biomedical ethics principles (Beauchamp and Childress, 2013). First, this approach facilitates equity in healthcare access: evidence-based healthcare for all. Then, as previous studies have highlighted both the effectiveness and harmlessness of the treatments in question, professionals are certain to respect beneficence and non-maleficence. Finally, since patients’ choices cannot be followed without full information first and consent later, EBP is also responsible for respecting patients’ autonomy.

### For patients, receiving the best healthcare

Without reiterating here all the beneficial aspects that EBP provides for patients, a few key points should be enumerated. First, knowing that the healthcare professional one may face knows and uses the best evidence is extremely comforting for patients and constitutes probably one of the best ramparts against quackery. Moreover, as patient choices assume an important role, EBP respects the patients’ right to information, free will, and autonomy (Greek, *autos*: oneself and *nomos*: law), in the Kantian sense of the word and of his “*sapere aude*!” (dare to know) (Kant, 1991).

This new “epistemic share of knowledge between caregiver and care receiver” (Stiegler and Alla, 2022) transforms the therapeutic relationship, especially as patients, in addition to the information they receive from professionals, are also now much more informed through the internet. For musicians, considered as patients, the situation is positively changing: while they often express a lack of knowledge (Rousseau et al., 2020), this could progressively change.

As a result of a second paradigm change concurrent to EBP, namely the emergence of a biopsychosocial model in place of the biomedical one, we now know to take care of our patients much more holistically, considering not only their physiological situation but the social and physiological factors may influence their health.

### Evidence-based practice on a social scale

Incorporating the highest evidence in our clinical practice serves to be beneficial for humanity and bodes well for social justice. According to utilitarians but also to John Rawls in his *Theory of Justice* (Rawls, 2005), it is our duty to fight against injustice and inequality [since it cannot be considered as a “fact of nature” (Spitz, 2011)], in this context unequal healthcare access. Theoretically, our French healthcare system entitles all individuals to the best healthcare, most of them being refunded by national solidarity, regardless of their life or work habits. Smoking does not mean that someone’s cancer will not be treated using the best level of evidence or cost more compared to someone who has never touched a cigarette. Having chosen to become an orchestra musician does not mean that they may be considered responsible for work-related ear issues.

Moreover, in its more recent definitions, the concept of healthcare resources has been added to EBP (Rogers and Hutchison, 2017), considering the sanitary context. Together, EBP and *New Public Health* inform the QUANGOs (QUasi Non-Governmental/QUasi-Autonomous National Government Organisations) that disseminate guidelines and assess practices. However, it should be mentioned that these recommendations are often designed for very frequent pathologies, not considering specific cases such as musicians’ common injuries.

## Part II: evidence-based practice, between straightjacket and pipe dream

### Combining clinic and research: does the “healthcare practitioner-researcher” exist?

While the evidence-based approach strives provide the best healthcare, it is highly complex. The question remains: how can we adapt the protocols of RCTs, which are carried out on drastically different populations to our patients? And how do we cope with the *grey zones*, “where the evidence about risk–benefit ratios of competing clinical options is incomplete or contradictory,” that we particularly face with musicians (Naylor, 1995)? How do we answer our clinical questions to tackle focal dystonia when evidence is lacking?

Also, although the theoretical borders of EBP have been thoroughly described, articulating the three circles of the triptych is still left to one’s interpretation. Moreover, integrating the highest evidence level in clinical practice requires donning the researcher’s three-piece costume: find, read, and appraise. But to put it on, you have to own it, and many barriers to EBP have been highlighted in literature. Such barriers include reading in English (for non-native speakers), mastering scientific methods (i.e., statistical tools, methodological bias) or simply having access to literature and being able to perform searches (Da Silva et al., 2015). For example, it is essential to have access to peer-reviewed journals such as the *Medical Problems of Performing Artists* journal in order to apprehend a great number of issues about musicians’ health. It would be dishonest not to mention the dramatic time-consuming nature of reading papers, when most of healthcare practitioners already experience the downward spiral of their entangled professional and personal lives.

The RCT, being at the top of the evidence hierarchy in EBP, provokes questioning. RCTs have been criticized for several reasons, including their mediocre external validity (and an excellent internal validity), therefore limited applicability to real-world practice, their inadequacy to certain practices such as physiotherapy (contrariwise to pharmaceutical trials), and their ignorance of the *black box* and the contextual elements of interventions (Rogers and Hutchison, 2017). For example, if prevention programs for playing-related musculoskeletal disorders may have a significant effect on performance or on injury prevalence, this does not answer the following question: why they do have an effect? And in the context of musicians, it is difficult to conduct an adequately powered trial due to the limited number of participants. This leads researchers toward conducting epidemiological studies, the results of which must be considered with extreme caution.

Limited samples are especially an important issue in studies of risk factors. Indeed, few existing systematic reviews (Baadjou et al., 2016; Kok et al., 2016) have concluded that there is too much heterogeneity in the data among primary studies, whether regarding the populations of musicians studied (professionals, students, amateurs) or the used definition of PRDMs. This led other research teams to work on risk factors in a different way, in order to describe them while being very cautious about how to interpret their significance in the development of these disorders. Chan and Ackermann (2014) in their literature review provide details about non-modifiable and modifiable risk factors. Rousseau et al. (2021) in their study combining narrative review and interviews (conducted with orchestra musicians and specialized healthcare professionals) provide a comprehensive theoretical model describing nine categories (individual characteristics, posture, biomechanics, injury management, workload, physical activity, life habits, environment and psycho-social factors). Current research is already evolving the definition of PRMDs (Zaza et al., 1998), moving toward a more comprehensive definition of “performance-related pain” (Zão et al., 2024) and an associated questionnaire, which may help researchers to better investigate risk factors and provide brighter comparisons between their different works.

### Patient of the *Lumières*: the “know and decide” injunction

Although the first part describes how patients could endorse the role of the “patient of the *Lumières*” (referring to the eighteenth-century Age of Enlightenment), informed and deciding for himself, it should be noted the place from which the patient speaks. As Susan Sontag has written, “illness is the night-side of life, a more onerous citizenship” (Sontag, 1978). Talcott Parsons has theorized the moral obligation of being a “good patient” in the eyes of society. Thus, although solidly informed, patients may struggle in formulating their own choices and decisions – “*sapere aude*!” (Kant, 1991) is a difficult injunction to satisfy. Moreover, is it reasonable to expect that scientific literature search and reading, already a difficult task for healthcare professionals, can be easily and adequately performed by our patients? Despite all our goodwill, it is almost impossible to erase this asymmetrical power that resides in the therapeutic relationship, as emphasized by Foucault. This brings to mind an anecdote: the situation of Rémi, a 14-year-old violinist, striving to become a professional musician. Suffering from an injury to his right wrist for several months, his rheumatologist, after having recommended to him to stop instrumental practice first for 2 weeks, then 1 month, advised him to stop playing the violin and change to another instrument. However, after a few weeks of postural rehabilitation and strengthening exercises, Rémi was able to play to pre-injury levels. What exactly happened between Rémi and his rheumatologist which almost resulted in the sudden break of a career that had barely begun?

Finally, since patients are the ontological object of research, it seems important to mention the crucial role that patients themselves could play in the development of trial protocols to identify and answer the clinical questions that are most meaningful to them. This integration of the patient perspective into study design leads to identification of cellists as musicians who would like to play on adaptable chairs (and thus evaluate the effects on their posture and performance) or orchestra musicians as ones who would like to have their own stand instead of sharing it. Example is research done by Spahn et al. (2014) in which standing and sitting position have been investigated in upper string players (*n* = 16) using posturography and 3D motion analysis. Results show that musicians report a preference for the standing position rather than the sitting position, reporting better weight distribution while standing, as well as greater freedom of movement. However, this does not reflect their professional daily life in the orchestra, as instrumentalists play most of the time seated. Spahn et al. (2014) highlighted that being seated on the right or on the left of the music stand impacted the weight distribution in a contralateral way (e.g.: loading the right side more than the left one while sitting on the left side of the stand). Although these conclusions should be considered with caution, they are interesting for reconsidering the working environment of musicians and collaborating with them to maximize the prevention of PRMDs.

### Globalization and control: evidence-based practice to the test of society

Some of the major promoters of EBP, including John Ioannidis, claim it has been “hijacked” (Ioannidis, 2016). A vivid example of this is the pharmaceutical bias, which is still often overlooked and poorly corrected. A clinical trial funded by the pharmaceutical industry will be three to four times more likely to be published than its public counterpart. Also, Ioannidis warns us about the use of big data in research which, according to him, could gradually overwhelm the scientific method.

Evidence is also a political, cultural, and social entity (Goldenberg, 2010), and we need to bear in mind the “power relationships internal to the world of scholars” which Bernard Lahire (French sociologist), while prefacing and commenting Norbert Elias’ work (German sociologist), describes as both “cooperation and competition relationships” (Lahire, 2016).

Mirroring the evidence crisis, the clinic faces tremendous challenges. As described by Stéphane Velut in his essay “Hospital, a new industry,” the clinic is flooded by consulting firms (Velut, 2020) and finance-based medicine (Ioannidis, 2016), converting the stock-hospital to a flow-hospital. It must be acknowledged that musicians do not represent a large “market share.”

Finally, it appears particularly relevant for musicians, whose professional lives vary considerably from one to another, that ignoring social factors while assessing health in individuals (which is much more difficult to investigate and evaluate compared to psychological factors) opens the door to potential therapeutic failure. As an example, advising orchestra musicians to increase their breaks number and duration could be considered as a tremendous idea from a physiological standpoint and it is often advocated (Chan and Ackermann, 2014), but it is unrealistic in practice when you have to rehearse and perform with both the orchestra and conductor.

## Part III: evidence-based practice, a worthwhile approach

In this last part, few hypotheses will be discussed to remedy the previously mentioned challenges in the EBP of musicians.

### Values-based practice

First, we would like to consider the *values-based practice* as our primary hypothesis (Fulford, 2004). This concept does not reject scientific evidence; instead, it emphasizes that many therapeutic failures stem from value conflicts and that it is essential to view the clinical practice as an alliance between evidence and values. Fulford, an American psychiatrist, highlighted language and communication as mediums through which we can better understand patients’ values and encompass those of healthcare professionals. A *values-based practice* shows great promise in facilitating better dialog between these two perspectives, which often struggle to comprehend each other. For example, injured musicians often delay seeking help because they fear being told to interrupt their instrumental practice (Stanhope and Weinstein, 2021).

### Reconsider the psychosocial factors

As previously mentioned, the well-named biopsychosocial model (Engel, 1977) attempts to merge the biological causes of diseases with the psychological and social factors that can influence their onset or chronification. However, while psychological factors are very often investigated in research and assessed in the clinic, social ones do not share the same fate, despite their undeniable importance. We are convinced that social factors are critical, but in most cases, it is impossible to take any action to alter them. Consider the orchestra musician: apart from his personal practice, nothing falls within his personal agency – in his position in the orchestra or his practice breaks, there is no room for manoeuver. The same can be said of other professions such as home support or delivery workers and this heart-breaking “Sorry we missed you” by Ken Loach, in Manchester suburbs (Sorry we missed you, 2019).

### Give clinical expertise its due

Finally, healthcare practitioners and researchers face a great challenge: the imperative to reintegrate clinical expertise. It appears that conducting qualitative studies – that is to say, exploring life experiences, emotions and feelings of both patients and professionals – may contribute to be achieve success in this endeavor. This approach has been undertaken several times in specialized literature in musicians’ health, such as studies investigating the lived experience of musicians with playing-related injuries (Guptill, 2011) or representations of body and health (Schoeb and Zosso, 2012). In both these studies, attention is focused on the individual experiences of musicians: one concerning their life with their injury and their often difficult care journey (Guptill, 2011), the other on their awareness (or lack thereof) of the importance of the human body in serving instrumental performance (Schoeb and Zosso, 2012). Questioning individuals on their proper life allows one to refute the mind–body dualism, as both Dewey (1916) or Canguilhem (1966) have done previously, to travel to the ends of the biopsychosocial model, and to capture all the aspects of the “vicissitudes of life.”

## Conclusion

According to the EBM working group’s explicit notion that “the new paradigm puts a much lower value on authority,” it may appear medical paternalism is vanishing. However, one form of authority must not hide another, and the use of evidence must not become a dogma. When treating musician patients, while research is still scarce and methods sometimes unsatisfactory, physiotherapists and other specialized healthcare professionals may often find themselves navigating across the *grey zones* of clinical practice (Naylor, 1995). This paper highlights the importance of conducting further research on musicians’ health and well-being, as it is supported by reviews and clinical trials, aforementioned in this manuscript, but also the meaning and significance of being *uncertain* while facing specific disorders or health conditions. Both uncertainty and autonomy, along with the possibility of therapeutic failure, are philosophical concepts that merit more extensive discussion in the initial training of healthcare professionals, particularly when in the context of caring for underrepresented populations in current research.

## Author contributions

CR: Writing – original draft, Writing – review & editing. BS: Writing – review & editing, Methodology, Supervision.

## References

[ref1] Baadjou V. Roussel N. Verbunt J. A. Smeets R. J. E. M. de Bie R. A. (2016). Systematic review: risk factors for musculoskeletal disorders in musicians. Occup. Med. 66, 614–622. doi: 10.1093/occmed/kqw052, PMID: 27138935

[ref2] Beauchamp T. L. Childress J. F. (2013). Principles of biomedical ethics. 7th Edn. New York: Oxford University Press.

[ref3] Bizouarn P. (2019). Evidence-based medicine et expertise clinique. Multitudes n° 75, 103–113. doi: 10.3917/mult.075.0103

[ref4] Canguilhem G. (1966). Le Normal et le Pathologique. Paris: Presses Universitaires de France.

[ref5] Chan C. Ackermann B. (2014). Evidence-informed physical therapy management of performance-related musculoskeletal disorders in musicians. Front. Psychol. 5:706. doi: 10.3389/fpsyg.2014.00706 25071671 PMC4086404

[ref6] Da Silva T. M. Costa L. D. C. M. Garcia A. N. Costa L. O. P. (2015). What do physical therapists think about evidence-based practice? A systematic review. Man. Ther. 20, 388–401. doi: 10.1016/j.math.2014.10.009 25458142

[ref7] Davidoff F. Haynes B. Sackett D. Smith R. (1995). Evidence based medicine. BMJ 310, 1085–1086. doi: 10.1136/bmj.310.6987.1085, PMID: 7742666 PMC2549494

[ref8] Dewey J. (1916). Démocratie et Éducation. Paris: Armand Colin, 1990.

[ref9] Evidence-Based Medicine Working Group (1992). Evidence-based medicine: a new approach to teaching the practice of medicine. JAMA 268, 2420–2425. doi: 10.1001/jama.268.17.2420 1404801

[ref10] Engel G. (1977). The need for a new medical model: a challenge for biomedicine. Science 196, 129–136. doi: 10.1126/science.847460, PMID: 847460

[ref11] Foucault M. (2003). Naissance de la clinique. Paris: Presses Universitaires de France.

[ref12] Fulford B. K. M. W. (2004). “Ten principles of values-based medicine”, in Schramme. Thomas & Thome, Johannes, Philosophy and Psychiatry, Berlin: Walter de Gruyter, 50.

[ref13] Garcia A. N. Costa L. D. C. M. De Souza F. S. De Almeida M. O. Araujo A. C. Hancock M.. (2018). Reliability of the mechanical diagnosis and therapy system in patients with spinal pain: a systematic review. J. Orthop. Sports Phys. Ther. 48, 923–933. doi: 10.2519/jospt.2018.7876, PMID: 29932871

[ref14] Goldenberg M. (2010). Perspectives on evidence-based healthcare for women. J. Women's Health 19, 1235–1238. doi: 10.1089/jwh.2009.1680 20575678

[ref15] Guptill C. A. (2011). The lived experience of professional musicians with playing-related injuries: a phenomenological inquiry. Med. Probl. Perf. Art. 26, 84–95. doi: 10.21091/mppa.2011.2013, PMID: 21695356

[ref16] Haynes R. B. Devereaux P. J. Guyatt G. H. (2002). Clinical expertise in the era of evidence-based medicine and patient choice. BMJ Evid. Based Med. 7, 36–38. doi: 10.1136/ebm.7.2.36 12749371

[ref17] Hill A. B. (2011). La philosophie de l’essai clinique (1962), in Gaille, Marie, Philosophie de la Médecine. Frontières, savoir, clinique, Paris: Vrin.

[ref001] Howick J. Chalmers I. Glasziou P. Greenhalgh T. Heneghan C. Liberati A.. (2011). The 2011 Oxford CEBM levels of evidence (Introductory Document).

[ref18] Ioannidis J. P. (2016). Evidence-based medicine has been hijacked: a report to David Sackett. J. Clin. Epidemiol. 73, 82–86. doi: 10.1016/j.jclinepi.2016.02.012, PMID: 26934549

[ref19] Kant E. (1991). Vers la paix perpétuelle. Que signifie s’orienter dans la pensée? Qu’est-ce que les Lumières? Paris: GF Flammarion.

[ref20] Kok L. M. Huisstede B. M. Voorn V. M. Schoones J. W. Nelissen R. G. (2016). The occurrence of musculoskeletal complaints among professional musicians: a systematic review. Int. Arch. Occup. Environ. Health 89, 373–396. doi: 10.1007/s00420-015-1090-6, PMID: 26563718 PMC4786597

[ref21] Kuhn T. (1983). La structure des révolutions scientifiques. Paris: Flammarion.

[ref22] Lahire B. (2016). « Préface » dans Elias, N., La dynamique sociale de la conscience. Paris: Editions La Découverte.

[ref23] Lemoine M. (2011). La désunité de la médecine – Essai sur les valeurs explicatives de la science médicale. Paris: Editions Hermann.

[ref24] Levenderis F. Rennie-Salonen B. (2022). Musculoskeletal symptoms of double bassists: a literature synthesis. Online J. Bass Res:14.

[ref25] McCrary J. M. Halaki M. Ackermann B. J. (2016). Effects of physical symptoms on muscle activity levels in skilled violinists. Med. Probl. Perf. Art. 31, 125–131. doi: 10.21091/mppa.2016.3024, PMID: 27575287

[ref26] Naylor D. (1995). Grey zones of clinical practice: some limits to evidence-based medicine. Lancet 345, 840–842. doi: 10.1016/S0140-6736(95)92969-X, PMID: 7898234

[ref28] Rawls J. (2005). A theory of justice (1971). Cambridge (Massachussetts): Belknap Press of Harvard University Press.

[ref29] Rogers W. Hutchison K. (2017). “Evidence-based medicine in theory and practice: epistemological and normative issues” in Handbook of the philosophy of medicine, Dordrecht. eds. Schramme T. Edwards S. (Dordrecht: Springer).2017

[ref30] Rousseau C. Del Valle Acedo S. Martin S. (2020). Troubles musculosquelettiques liés à l’exécution musicale chez l’étudiant en jazz et musiques improvisées: une étude qualitative. Kinésitherapie La Revue 20, 2–8. doi: 10.1016/j.kine.2020.04.004

[ref31] Rousseau C. Barton G. Garden P. Baltzopoulos V. (2021). Development of an injury prevention model for playing-related musculoskeletal disorders in orchestra musicians based on predisposing risk factors. Int. J. Ind. Ergon. 81:103026. doi: 10.1016/j.ergon.2020.103026

[ref32] Sackett D. L. Rosenberg W. M. C. Gray J. A. M. Haynes R. B. Richardson W. S. (1996). Evidence based medicine: what it is and what it isn't. BMJ 312, 71–72. doi: 10.1136/bmj.312.7023.71, PMID: 8555924 PMC2349778

[ref33] Schoeb V. Zosso A. (2012). “You cannot perform music without taking care of your body”: a qualitative study on musicians’ representation of body and health. Med. Probl. Perf. Art. 27, 129–136. doi: 10.21091/mppa.2012.3024 22983130

[ref34] Sontag S. (1978). Illness as metaphor. New York: Farrar, Strauss and Giroux.

[ref35] Sorry we missed you (2019). Directed by ken loach. Paris: Why Not Productions.

[ref36] Spahn C. Wasmer C. Eickhoff F. Nusseck M. (2014). Comparing violinists’ body movements while standing, sitting, and in sitting orientations to the right or left of a music stand. Med. Probl. Perf. Art. 29, 86–93. doi: 10.21091/mppa.2014.2019, PMID: 24925176

[ref37] Spitz J. F. (2011). John Rawls et la question de la justice sociale. Études Tome 414, 55–65. doi: 10.3917/etu.4141.0055

[ref38] Stanhope J. Weinstein P. (2021). Should musicians play in pain? Br. J. Pain 15, 82–90. doi: 10.1177/2049463720911399, PMID: 33633855 PMC7882775

[ref39] Stiegler B. Alla F. (2022). Santé publique année zéro. Paris: Tracts Gallimard.

[ref40] Tawde P. Dabadghav R. Bedekar N. Shyam A. Sancheti P. (2016). Assessment of cervical range of motion, cervical core strength and scapular dyskinesia in violin players. Int. J. Occup. Saf. Ergon. 22, 572–576. doi: 10.1080/10803548.2016.1181892, PMID: 27232160

[ref41] Valenzuela-Gómez S. A. Rey-Galindo J. A. Aceves-Gonzlez C. (2020). Analyzing working conditions for classical guitarists: design guidelines for new supports and guitar positioning. Work 65, 891–901. doi: 10.3233/WOR-203140, PMID: 32310218

[ref42] Velut S. (2020). L’hôpital, une nouvelle industrie. Paris: Tracts Gallimard.

[ref43] Zão A. Altenmüller E. Azevedo L. (2024). Performance-related pain among musicians questionnaire (PPAM): multicenter validation of the first questionnaire to evaluate performance-related pain among musicians with different musical backgrounds. J. Pain 25, 393–406. doi: 10.1016/j.jpain.2023.09.003, PMID: 37690474

[ref44] Zaza C. Charles C. Muszynski A. (1998). The meaning of playing-related musculoskeletal disorders to classical musicians. Soc. Sci. Med. 47, 2013–2023. doi: 10.1016/S0277-9536(98)00307-4, PMID: 10075243

